# Comparative cadaveric biomechanical analysis of vastus medialis advancement and medial patellofemoral ligament reconstruction

**DOI:** 10.1186/s13018-026-06743-1

**Published:** 2026-03-17

**Authors:** Cihad Çağrı Üstün, Semih Aydoğdu, Elçil Kaya Biçer, Buğra Hüsemoğlu, Abdullah Faruk Uyanık

**Affiliations:** 1 Department of Orthopaedics and Traumatology, Ege University School of Medicine, Izmir, Turkey; 2 Department of Biomechanics, Dokuz Eylül University, Izmir, Turkey; 3 Department of Orthopaedics and Traumatology, Okmeydanı Training and Research Hospital, Istanbul, Turkey

**Keywords:** Medial patellofemoral ligament reconstruction, Vastus medialis advancement, Lateral patellar instability, Cadaveric biomechanical study, Knee surgery

## Abstract

**Purpose:**

Restoration of medial soft-tissue restraint is essential in the surgical treatment of lateral patellofemoral instability. While anatomic medial patellofemoral ligament reconstruction (MPFLR) has become the preferred technique, non-anatomic procedures such as vastus medialis advancement (VMA) are still used in selected clinical scenarios. However, controlled biomechanical data comparing these techniques remain limited. This study aimed to evaluate and compare the tensile behavior of VMA and MPFL reconstruction using a cadaveric biomechanical model.

**Methods:**

Ten fresh-frozen human cadaveric knees were mounted in a custom-designed biomechanical testing apparatus that simulated physiological quadriceps loading. Progressive lateral force was applied to reproduce patellar dislocation, and the failure load of the native medial patellofemoral ligament was recorded. Specimens were then randomized into two groups: MPFL reconstruction (*n* = 5) and vastus medialis advancement (*n* = 5). Tensile testing was repeated following each procedure, and the forces required to produce 10, 20, 30 and 40 mm of lateral patellar displacement were measured and analyzed.

**Results:**

Following reconstruction, the MPFLR group demonstrated numerically higher tensile force values at 10, 20, and 30 mm of lateral patellar displacement compared with the native condition, whereas the VMA group exhibited lower tensile force values across this physiologically relevant displacement range. At 40 mm displacement, which exceeds physiological patellar translation and reflects failure behavior rather than functional stability, a reduction in tensile force was observed in both groups. Overall, mean tensile force values tended to be higher in the MPFLR group than in the VMA group; however, no statistically significant differences were observed between the two techniques in either the medial soft-tissue injury induction test or the post-reconstruction tensile rupture test (*p* > 0.05 for all comparisons).

**Conclusion:**

In this cadaveric biomechanical study, medial patellofemoral ligament reconstruction and vastus medialis advancement demonstrated different construct behavior patterns, with no statistically significant differences in the tensile force required to achieve lateral patellar displacement.

## Introduction

Patellofemoral instability represents a complex disruption of the balance within the patellofemoral joint, frequently resulting in recurrent dislocation, anterior knee pain, and functional impairment. It commonly occurs after rotational trauma in individuals with generalized ligamentous laxity or predisposing anatomic abnormalities such as trochlear dysplasia, patella alta, increased tibial tubercle–trochlear groove (TT–TG) distance, excessive femoral anteversion, tibial external rotation, and muscular imbalance [1]. Acute lateral patellar dislocation typically involves disruption of the MPFL and may also injure other medial soft tissues, reflecting the complexity of medial restraint mechanisms [2]. A comprehensive understanding of these pathoanatomical factors is essential for selecting the appropriate surgical treatment strategies [3]. Distal realignment procedures such as Elmslie-Trillat, Fulkerson, Maquet, and Roux-Goldthwait yield low recurrence rates in the management of patellar dislocation [4]. Following first-time patellar dislocation, MPFL rupture occurs in nearly all cases and is often accompanied by chondral injury, indicating the fundamental role of the medial soft-tissue restraints in maintaining patellofemoral stability [5].

The medial patellofemoral ligament is widely recognized as the primary soft-tissue stabilizer resisting lateral patellar displacement. Injury to the medial stabilizing structures—including the MPFL, medial retinaculum, and medial patellotibial ligament (MPTL)—is commonly observed during lateral dislocation events [6, 7]. Although the MPTL has been anatomically described as a distinct stabilizer potentially contributing to patellar rotational control, its specific role in resisting lateral translation remains incompletely characterized [7]. Since its description as a distinct anatomical structure in 1979, the MPFL has been consistently identified in the majority of knees, supporting its role in patellofemoral stability [8].

Both conservative and surgical strategies have been employed in the management of patellar instability. While conservative treatment of patellar dislocation primarily focuses on exercise-based stabilization, surgical approaches aim to restore medial soft-tissue restraint in patients with recurrent instability [9]. Medial patellofemoral ligament reconstruction (MPFLR) has become a widely adopted anatomic procedure, with numerous clinical studies reporting favorable functional outcomes and reduced redislocation rates across various graft and fixation techniques [10–17].

Combined MPFL and MPTL reconstruction has been associated with favorable functional outcomes and low recurrence rates in recurrent patellofemoral instability [18, 19]. Nevertheless, the surgical approach must be tailored to individual anatomic variations, as MPFL insufficiency often coexists with other predisposing factors [20, 21].

Before the widespread adoption of MPFLR, various non-anatomic soft-tissue realignment procedures such as vastus medialis advancement (VMA) were utilized. VMA involves overlapping the vastus medialis obliquus to augment medial restraint. Although initially associated with inconsistent outcomes, later studies reported improved results with technical modifications [22–24]. Both VMA and MPFL reconstruction aim to enhance medial patellar stability; however, their relative biomechanical behavior under controlled experimental conditions has not been clearly defined [12, 20, 25].

Despite extensive clinical literature, the transition from VMA to MPFL reconstruction has been guided primarily by clinical experience and consensus rather than direct biomechanical comparison. To date, no biomechanical study has directly compared the tensile force behavior of vastus medialis advancement and medial patellofemoral ligament reconstruction under standardized experimental conditions.

Therefore, the purpose of the present study was to compare the tensile force–displacement behavior of VMA and MPFL reconstruction using a human cadaveric biomechanical model.

## Methods

### Study design and aim

This controlled laboratory study aimed to compare the tensile properties of medial patellofemoral ligament reconstruction (MPFLR) and vastus medialis advancement (VMA) using human cadaveric knees. All experiments were performed at a certified biomechanical research facility under standardized laboratory conditions.

### Specimens

Ten fresh-frozen human cadaveric lower extremities (six female and two male donors), extending from the mid-thigh to the toes, were obtained through Interra Sağlık (Ankara, Turkey) in collaboration with Science Care (Phoenix, USA). Four knees were obtained bilaterally from two donors, while the remaining six knees were obtained unilaterally from six different donors. In cases of bilateral specimens, each knee was assigned to a different experimental group to minimize intra-donor dependency. Specimen allocation was determined by availability, as donor selection and laterality could not be influenced due to the institutional procurement process. Although bilateral specimens were available in two donors, the study did not involve fully matched left–right pairs, and no sequential or crossover procedures were performed on the same knee.

The mean age of the donors was 82.1 ± 8.2 years, mean body mass index 21 ± 3.4 kg/m^2^. The mean donor height was 1.62 m, and the mean body weight was 56.1 ± 13.1 kg. No specimen showed evidence of prior surgery, deformity, trauma, or osteoarthritis.

Each pair of matched specimens was randomly assigned to either the MPFLR group (*n* = 5) or the VMA group (*n* = 5). All procedures complied with institutional ethical standards and cadaveric research regulations (approval no. 70198063-050.06.04).

### Specimen preparation

Specimens were maintained under cold-chain conditions and stored at –18 °C. Forty-eight hours before testing, they were thawed gradually—first at +4 °C for 24 hours, then at room temperature while wrapped in saline-soaked gauze to prevent desiccation. The skin and subcutaneous tissues were removed while preserving capsular and periarticular soft-tissue structures. Femora and tibiae were cut approximately 15 cm from the joint line and fixed in cylindrical holders to ensure stable mounting.

### Biomechanical testing setup

**Fig. 1:**
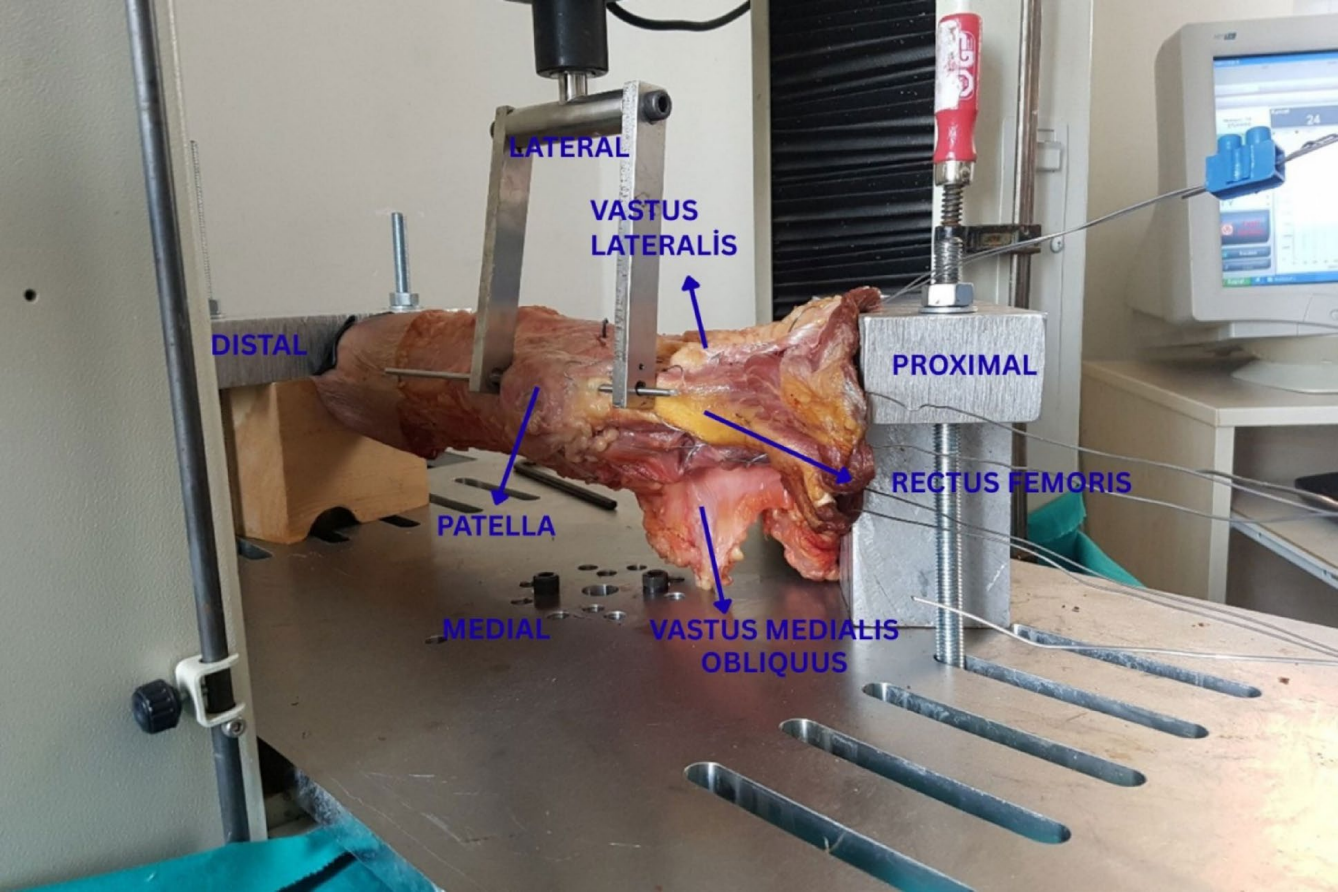
A right knee specimen is shown, rotated 90° to the left to allow a lateral view of the patellofemoral joint. The knee was positioned at 30° of flexion throughout testing. Force vectors corresponding to the vastus medialis (VM), vastus lateralis (VL), and rectus femoris (RF) are indicated with arrows. The Steinmann pin inserted into the lateral third of the patella and the direction of applied lateral displacement are also shown

A custom-built biomechanical testing apparatus was mounted on a servo-hydraulic testing system (Shimadzu AG-IS 5 kN, Kyoto, Japan) to quantify the force required for lateral patellar displacement. The experimental setup and knee positioning are illustrated in Fig. 1.

**Fig. 2:**
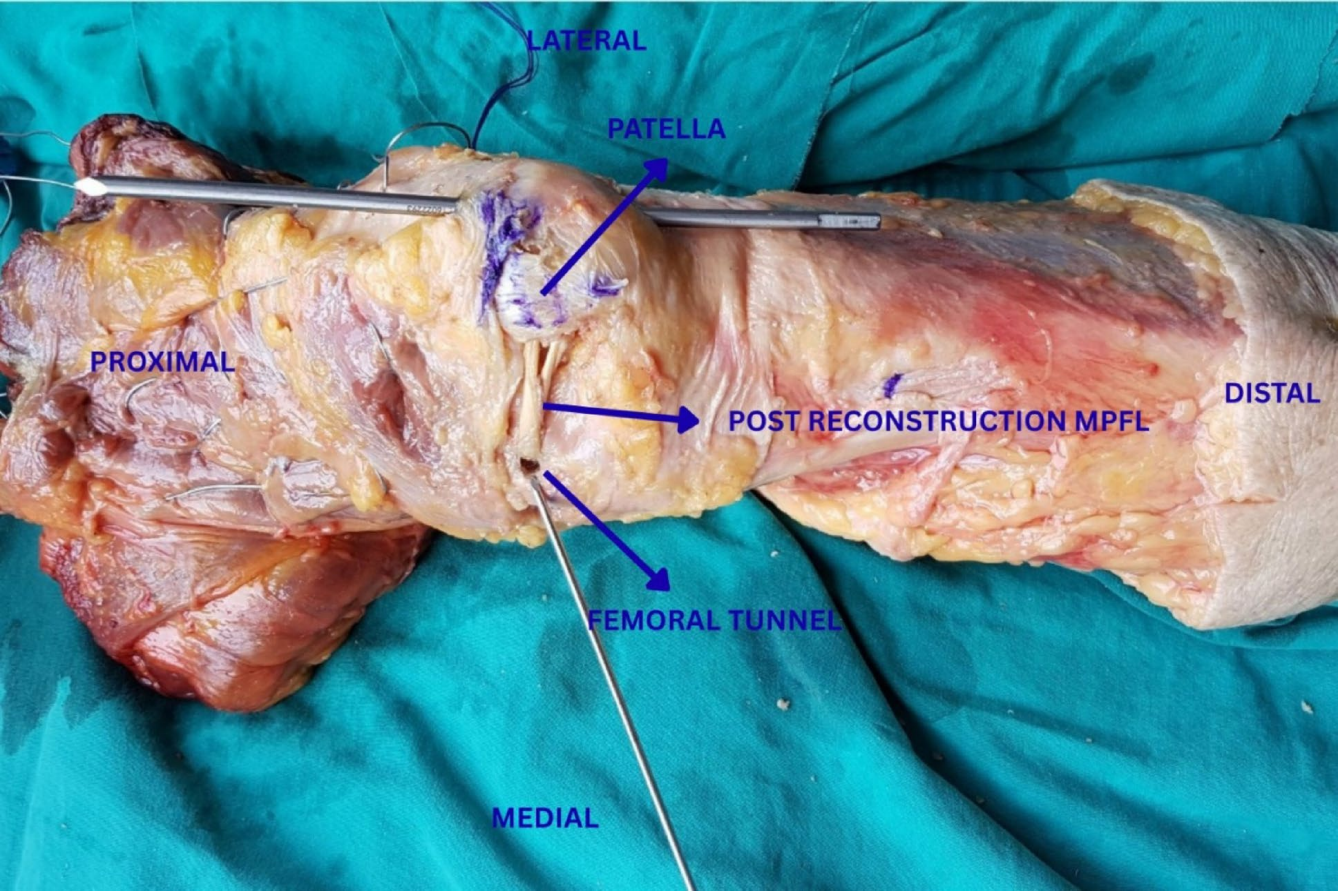
Femoral and patellar tunnel locations used for medial patellofemoral ligament reconstruction in the cadaveric knee specimens

The device replicated physiological quadriceps loading vectors with the knee fixed at 30° of flexion. A 5-mm Steinmann pin was inserted longitudinally along the sagittal axis of the patella, through its lateral third, as shown in Figs. 1 and 2, and cerclage wires were placed around the quadriceps tendon using the Krackow technique. The magnitude and direction of the applied forces were selected to simulate physiological patellofemoral loading.

Force vectors were aligned with the anatomical orientation of the quadriceps mechanism, corresponding to the lines of action of the vastus medialis, vastus lateralis, and rectus femoris. Force magnitudes were based on previously published cadaveric biomechanical studies on patellar stability.Simulated quadriceps forces were applied as follows: rectus femoris 60 N (0° mediolateral, 0° anteroposterior), vastus lateralis 57 N (14° lateral, 0° anteroposterior), and vastus medialis 40 N (47° medial, 44° posterior) [26].

Progressive lateral traction was then applied until 40 mm of displacement, producing rupture of the native medial soft-tissue structures. Force–displacement data were digitally recorded every 50 ms.

### Surgical procedures

#### MPFL reconstruction

The MPFL reconstruction technique used in this study was based on previously described and widely accepted surgical principles, as reported by Schöttle et al. and subsequent anatomical and biomechanical studies [27–29] A semitendinosus autograft was harvested, debrided, and prepared with Krackow sutures using absorbable braided suture material (Ethicon 1-0 Vicryl, Polyglactin 910).

Two 4.5 mm patellar tunnels were drilled medially to anteriorly in line with the tensile-testing axis. The femoral tunnel was created between the adductor tubercle and medial epicondyle at the Schöttle point using a 7 mm bit.

With the knee flexed at 60°, the doubled graft (final length, 115 mm) was passed through the patellar tunnels and fixed within the femoral tunnel using a titanium interference screw (TMIV-0925, 9 × 25 mm, Doratek, Istanbul, Turkey) [28]. During femoral fixation, the graft was tensioned manually to remove slack without over-constraint. Fixation was performed at 60° of knee flexion while confirming central patellar tracking throughout the range of motion. No dedicated tensiometer was used [30, 31].

The femoral and patellar tunnel locations for medial patellofemoral ligament reconstruction are illustrated in Fig. 2. Following fixation ;the joint was cycled through full flexion and extension ten times to confirm stability and graft integrity.

#### Vastus medialis advancement

A medial parapatellar incision was made over the vastus medialis obliquus (VMO) preserving a 2 mm tendon remnant. The incision extended 2 cm proximal to the upper pole of the patella down to the lower pole.

**Fig. 3:**
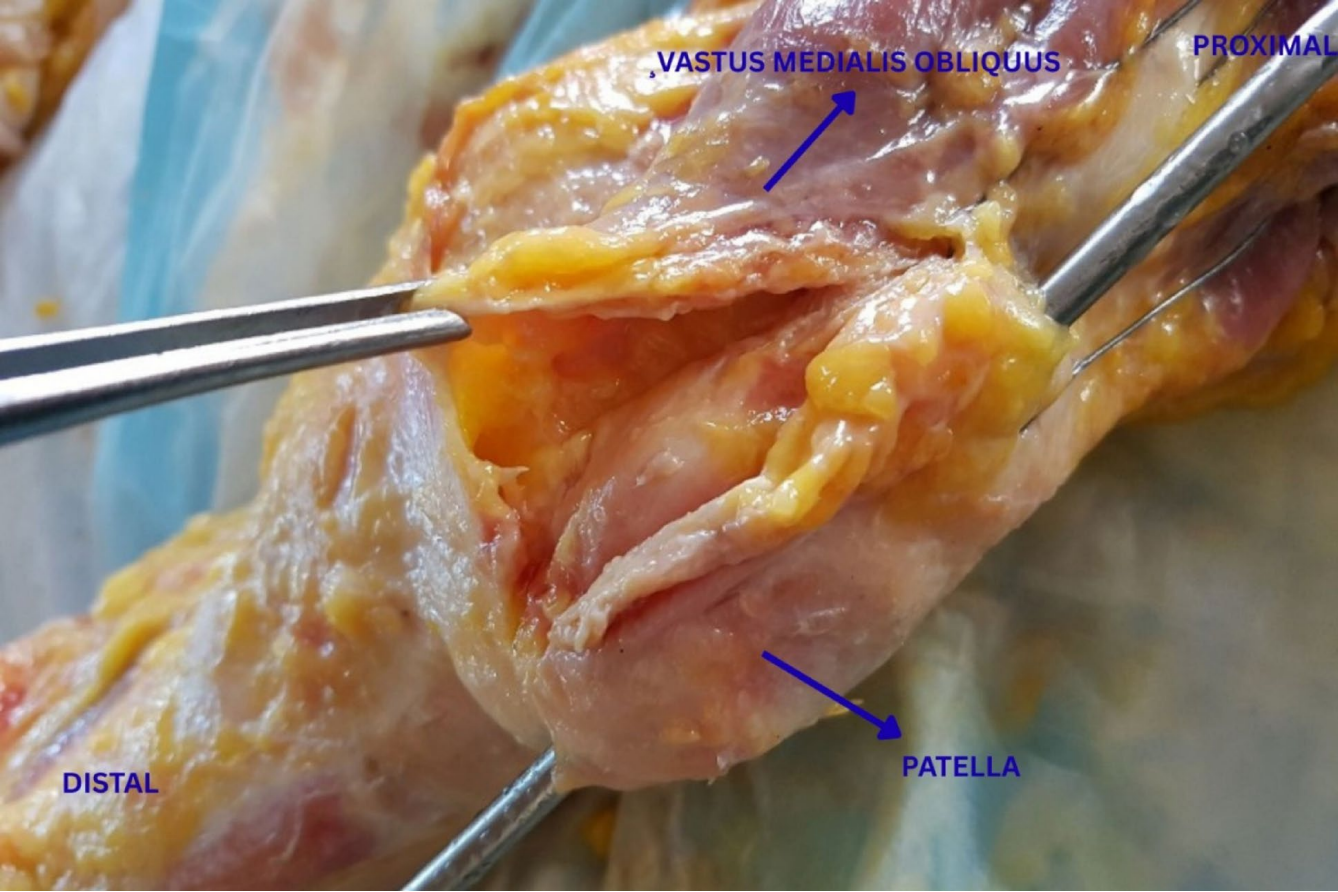
Intraoperative photograph illustrating the vastus medialis advancement (VMA) procedure performed on the cadaveric knee specimen

Two No. 2 FiberWire sutures (Arthrex, Naples, FL, USA) were placed 1 cm medial to the incision using a horizontal-mattress configuration, allowing the VMO to be advanced and overlapped by 1 cm. Sutures were tied under optimal tension at 45° knee flexion after ten flexion–extension cycles. Additional No. 1 Vicryl (Ethicon) reinforcement sutures were placed for stability. The vastus medialis advancement (VMA) procedure is illustrated in Fig. 3.

#### Post-reconstruction testing

After reconstruction, all specimens underwent identical lateral pull testing at 30° flexion. Tensile-rupture forces were recorded at 10, 20, 30 and 40 mm of lateral patellar displacement. The primary outcome was tensile force at each displacement interval; secondary outcomes included failure mode and comparison of pre- and post-reconstruction tensile properties within each group.

### Statistical analysis

The primary biomechanical outcome was the tensile force required to achieve predefined levels of lateral patellar displacement (10, 20, 30 and 40 mm). Tensile rupture force was recorded separately as a failure-related parameter.Statistical analyses were performed using IBM SPSS Statistics for Windows (version 21.0, IBM Corp., Armonk, NY, USA). Normality was tested using the Shapiro–Wilk test. Independent-samples Mann–Whitney U tests were used for non-parametric comparisons between groups. Within-group pre- and post-reconstruction differences were evaluated using paired t-tests or Wilcoxon signed-rank tests as appropriate. For repeated measures with more than two conditions, analysis of variance (ANOVA) or Friedman tests were applied, followed by Bonferroni or Wilcoxon post-hoc analyses. Statistical significance was set at *p* < 0.05. A formal power calculation was not feasible due to limited specimen availability; however, the sample size aligns with comparable cadaveric biomechanical studies of MPFL reconstruction [31–33].

## Results

Failure modes were recorded during tensile testing and analyzed descriptively. In the MPFL reconstruction group, failure predominantly occurred at the graft–fixation interface. In contrast, failure in the vastus medialis advancement group more commonly involved the muscle–tendon unit or suture loosening, reflecting differences in load transfer mechanisms between the two techniques. No consistent pattern of catastrophic bone failure was observed in either group.

During the medial soft-tissue injury induction tests at 10, 20, 30, and 40 mm of lateral displacement, the mean tensile forces in the MPFLR group were 85, 194, 215, and 350 N, respectively, whereas in the VMA group they were 86, 186, 319 and 446 N. No statistically significant differences were observed between groups at any displacement level (*p* > 0.05).Medial soft-tissue injury was not considered a single discrete event but rather a progressive process occurring with increasing lateral patellar displacement. Accordingly, tensile force values were recorded at predefined displacement levels during this progressive injury induction, prior to complete medial soft-tissue insufficiency.

Both groups demonstrated a progressive increase in tensile force with increasing displacement during the medial soft-tissue injury induction phase. Following reconstruction, distinct construct behavior patterns were observed between techniques within the physiologically relevant displacement range. The MPFLR group demonstrated numerically higher tensile force values at 10, 20 and 30 mm compared with measurements obtained at the corresponding displacement levels during progressive medial soft-tissue injury. At 40 mm displacement, which exceeds physiological patellar translation and reflects failure behavior rather than functional stability, a reduction in tensile force was observed. In contrast, the VMA group demonstrated lower tensile force values across displacement levels relative to the native state.

When comparing the two techniques after reconstruction, the MPFLR group exhibited numerically higher mean tensile force values at 10, 20, and 30 mm of lateral patellar displacement. At 40 mm displacement, exceeding physiological patellar translation and representing failure behavior rather than functional stability, the VMA group exhibited slightly higher force values; however, none of these differences reached statistical significance. (Table 1).

**Table 1:** Test results

Group	10 mm	20 mm	30 mm	40 mm
MPFLR Group				
Medial Soft Tissue Induction	85.47	193.59	215.00	350.42
Post-Reconstruction	117.34	225.46	274.37	280.31
*P* values (pre-post)	0.69	0.84	0.84	0.31
VMA Group				
Medial Soft Tissue Induction	85.63	186.25	319.22	445.50
Post-Reconstruction	74.69	147.03	246.09	310.94
*P* values (pre-post)	0.84	0.54	0.54	0.88
*P* values (medial soft tissue induction test between groups)	1	0.69	1	1
*P* values (post reconstruction test between groups)	0.42	0.84	1	0.41

Statistical comparisons did not demonstrate statistically significant differences between MPFLR and VMA in either the medial soft-tissue injury induction test (*p* = 1.000, 0.690, 1.000, and 1.000 at 10, 20, 30, and 40 mm, respectively) or the post-reconstruction tensile-rupture test (*p* = 0.421, 0.841, 1.000, and 0.413, respectively).

Although these differences did not reach statistical significance, MPFL reconstruction was associated with consistently higher tensile force values at 10, 20 and 30 mm of lateral patellar displacement compared with the native state. In contrast, VMA was associated with lower tensile force values across the physiologically relevant displacement range. At 40 mm displacement—exceeding physiological patellar translation and representing a failure condition rather than functional stability—both constructs demonstrated reduced tensile force values compared with measurements obtained during progressive medial soft-tissue injury.

**Fig. 4:**
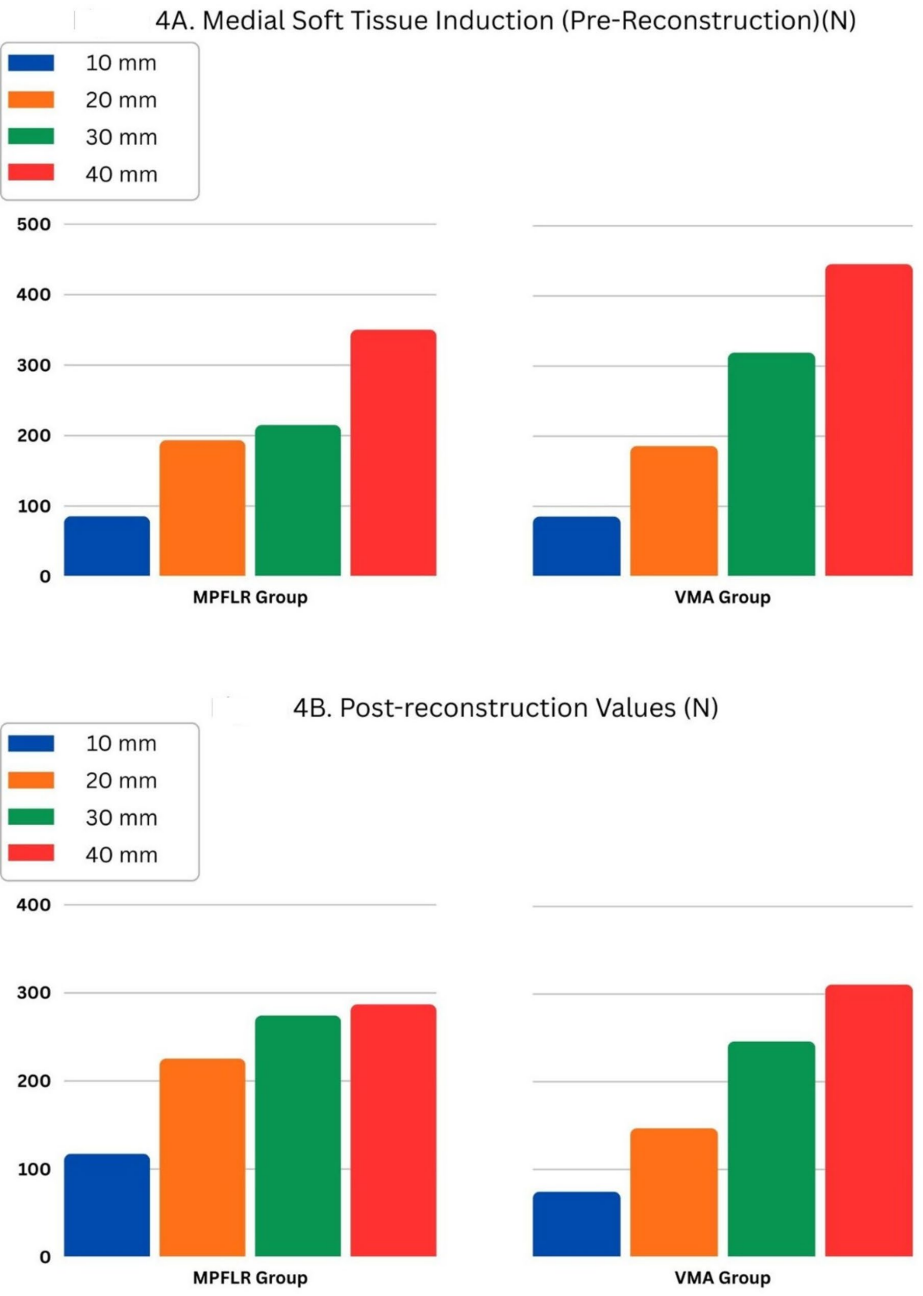
**A**: Tensile test data (N) following pre-reconstruction test for elongation between 10 and 40 mm. **B**: Tensile test data (N) following reconstruction for elongation between 10 and 40 mm

A summary of mean tensile forces at each displacement level is presented in Table 1. Figure 4A and B illustrate the force–displacement behavior and failure characteristics of both reconstruction techniques, highlighting the divergence in construct behavior beyond physiologically relevant displacement levels

No patellar fractures or other procedure-related complications were observed during the medial soft-tissue injury induction tests or the post-reconstruction tensile tests.

## Discussion

### Key findings

The principal finding of this study is that medial patellofemoral ligament reconstruction (MPFLR) and vastus medialis advancement (VMA) demonstrated comparable tensile force responses to lateral patellar displacement across the evaluated displacement levels, with no statistically significant differences between techniques. Although numerical variations in tensile force values were observed at lower displacement levels, these differences did not reach statistical significance and should be interpreted with caution. To our knowledge, this is the first biomechanical study to directly compare MPFLR and VMA within a controlled experimental framework. Accordingly, the findings should be interpreted primarily in a descriptive biomechanical context, focusing on construct behavior patterns rather than inferential comparisons.

### Original contribution

This study provides the first biomechanical quantification of the forces generated by vastus medialis advancement (VMA) against lateral patellar displacement. Following VMA, the force required to achieve 10, 20, 30, and 40 mm of lateral patellar translation was 74.69, 147.03, 246.09 and 310.94 N, respectively.

### Biomechanical interpretation

Current literature underscores the complexity of patellofemoral instability and the additive effect of multiple pathoanatomical factors on lateral displacement forces, highlighting the need for biomechanical analyses to supplement clinical outcome data [34]. In the current study, MPFLR and vastus medialis advancement demonstrated comparable tensile force responses to lateral patellar displacement up to 30 mm, which represents a physiologically relevant displacement range. At 40 mm displacement, exceeding physiological patellar translation and reflecting failure behavior rather than functional stability, a reduction in tensile force was observed following reconstruction. In a normally aligned knee, 40 mm of patellar translation is not physiologically achievable without structural disruption. These findings should therefore be interpreted as differences in load-sharing and construct behavior rather than indicators of biomechanical inferiority. The observed differences in failure patterns further support the concept that MPFL reconstruction and vastus medialis advancement exhibit distinct load-sharing mechanisms. Whereas MPFL reconstruction failures were primarily related to graft or fixation integrity, VMA failures tended to involve the muscle–tendon unit, which may be influenced by age-related muscle degeneration in cadaveric specimens.

Previous biomechanical studies have reported MPFL elongation or rupture at lateral displacements ranging between 8 and 34 mm [32, 35–37]. These findings are consistent with the present data, supporting that tensile testing beyond 30 mm represents non-physiological patellar dislocation. The advanced mean age of the cadaveric specimens (82 years) may also have contributed to increased tissue laxity compared with younger samples used in other studies [36, 38, 39].

There is currently no consensus on the optimal surgical approach for patellar instability. A Delphi study including surgeons from 11 countries demonstrated a lack of agreement regarding treatment preferences [40, 41] Systematic clinical data indicate that synthetic graft MPFL reconstructions improve functional scores and maintain low redislocation rates in recurrent patellofemoral instability [14]. Numerous techniques for MPFL reconstruction have been reported, differing in graft selection and femoral–patellar fixation methods. Using a semitendinosus autograft and a divergent two-tunnel configuration, Panni et al. demonstrated favorable functional outcomes and absence of postoperative redislocation in a prospective cohort of patients with recurrent patellar instability [42]. MPFL reconstruction using hamstring grafts through transverse patellar tunnels yields good functional results and low redislocation rates in recurrent instability [43].

Despite the extensive biomechanical research on fixation techniques, graft properties, and fixation angles in MPFLR, comparative biomechanical data evaluating MPFLR relative to vastus medialis advancement (VMA) remain unavailable. Current evidence suggests that MPFL reconstruction is clinically effective in reducing recurrent instability, but mechanistic, cadaver-based data are needed to clarify how different reconstruction or medialization techniques affect patellofemoral biomechanics [16] Clinical evidence indicates that hardware-free medial patellofemoral ligament reconstruction techniques yield significant improvements in functional outcomes and low redislocation rates in recurrent patellofemoral instability [44].

To our knowledge, no previous biomechanical study has quantified the tensile forces generated by vastus medialis advancement (VMA) against lateral patellar translation. This work offers the first comparative biomechanical evaluation of VMA relative to the native MPFL and MPFL reconstruction (MPFLR), providing new insight into the mechanical behavior of these stabilizing techniques.

Few clinical studies have directly compared the outcomes of MPFL reconstruction (MPFLR) and vastus medialis advancement (VMA). Malecki et al. reported comparable patellar stability following either technique [45]. In another study involving 69 patients, Wang et al. demonstrated superior outcomes when VMA was combined with MPFLR compared with MPFLR alone [46]. Clinically, VMA is commonly performed in conjunction with bony realignment procedures, including tibial tubercle medialization or distalization, particularly in cases with patella alta. Morrissey et al. found that the combination of tibial tubercle medialization, lateral retinacular release, and VMA reduced recurrent dislocation risk at 16-month follow-up [22]. Similarly, O’Beirne et al. reported that VMA was effective in managing recurrent patellar dislocations [23]. These findings indicate that VMA remains a viable soft-tissue procedure in selected patient populations.

In the present study, the forces required to laterally translate the patella with an intact MPFL were 85.54 N, 189.92 N, 267.11 N, and 365 N at 10, 20, 30, and 40 mm of displacement, respectively. These values are consistent with previously reported biomechanical findings. Bedi et al. measured 102.3 N at 10 mm of displacement [47], while Wang et al. and Tanner et al. reported 96.3 N at 10 mm and 84.43 N at 7.39 mm, respectively He et al. found that 146.91 N was required for an 8.39 mm elongation [35, 39, 48]. Systematic review data from Huber et al. indicated a mean maximum force of 158.3 N for 14.3 mm of translation [37]. Furthermore, Duchman et al. and Mountney et al. documented 69 N at 10 mm and 208 N at 26 mm of displacement, respectively [33, 36]. Taken together, these findings demonstrate that our baseline tensile force values closely align with published literature.

In this study, the forces required to laterally translate the patella following MPFL reconstruction (MPFLR) were 117.34 N, 225.46 N, 274.37 N, and 286.92 N at 10, 20, 30, and 40 mm of displacement, respectively. Reported values in the literature show similar results at 10 mm, including 92.1 N by Wang et al. following double-bundle MPFLR [39], 126 N and 195 N by Mountney et al. using two reconstruction techniques [36], and 110.2 N by Duchman et al. [33]. Although direct comparison is limited due to differences in surgical techniques and fixation configurations, our findings at low translation values appear consistent with previous studies. To our knowledge, biomechanical data describing tensile force at 20, 30, and 40 mm displacement after MPFLR have not been previously published, providing additional insight into the mechanical behavior of reconstructed MPFL across a wider range of lateralization.

The risk of recurrent patellar dislocation is higher in patients with open physes compared to skeletally mature individuals [49]. In such cases, surgical techniques that do not interfere with the physis and, consequently, growth should be preferred [49]. Within this context, vastus medialis advancement (VMA) is often prioritized. However, some surgeons advocate for MPFL reconstruction (MPFLR) in skeletally immature patients, reporting satisfactory long-term outcomes after 10 years [50]. Others suggest that physeal integrity can be preserved if tunnels are drilled at specific angles [51, 52].

### Limitations

This study has several limitations. The advanced mean age of the cadaveric specimens (82.1 years) represents a major limitation of this study. Age-related degeneration of soft tissues and muscle quality may have disproportionately affected the biomechanical performance of vastus medialis advancement (VMA). Therefore, the stabilizing effect of VMA observed in this study may be underestimated relative to younger patient populations.

Second, it was conducted on a limited number of cadaveric specimens (*n* = 10), which may restrict statistical power. Given the small sample size (*n* = 5 per group), this study was not powered to detect small between-group differences and should be considered exploratory in nature. Statistical analyses were therefore used descriptively to identify trends in force–displacement behavior rather than to establish definitive inferential conclusions.

Third, the experimental setup simulated only a single flexion angle (30°), and other dynamic variables—such as muscle activation and joint reaction forces—were not replicated.

The absence of a paired or crossover design represents a limitation of this study. Although a sequential design could theoretically reduce inter-specimen variability, MPFL reconstruction fundamentally alters medial soft-tissue anatomy and load-sharing characteristics, which would confound the biomechanical assessment of a subsequent VMA procedure. Therefore, each specimen underwent only one reconstruction technique.

Finally, all specimens were anatomically normal; thus, the findings may not fully represent clinical scenarios involving trochlear dysplasia or other pathoanatomic abnormalities.

## Conclusion

In this controlled cadaveric biomechanical study, medial patellofemoral ligament reconstruction and vastus medialis advancement demonstrated comparable tensile force responses to increasing lateral patellar displacement, with no statistically significant differences between techniques. Within the limitations of this experimental model, both procedures demonstrated similar biomechanical behavior restoring medial stabilizing function. Accordingly, these findings should be interpreted in a descriptive biomechanical context rather than as evidence of superiority of one technique over the other.

## Data Availability

The data that support the findings of this study are available from the corresponding author upon reasonable request.

## Abbreviations

MPFL: Medial patellofemoral ligament
MPFLR: Medial patellofemoral ligament reconstruction
MPTL: Medial patellotibial ligament
VMA: Vastus medialis advancement
VMO: Vastus medialis obliquus
TT–TG: Tibial tubercle–trochlear groove
ANOVA: Analysis of variance
SPSS: Statistical package for the social sciences
BMI: Body mass index
